# A Retrospective Analysis of Pregnancy Outcomes Following Frozen Embryo Transfer in Patients With Persistent Chronic Endometritis: A Five‐Year Single‐Center Study

**DOI:** 10.1002/iid3.70448

**Published:** 2026-04-20

**Authors:** Yihua Liang, Zhaoyue Huang, Haiyan Lin, Qi Qiu, Xuedan Jiao, Qingxue Zhang

**Affiliations:** ^1^ Reproductive Medicine Centre, Sun Yat‐sen Memorial Hospital Sun Yat‐sen University Guangzhou Guangdong China

**Keywords:** frozen embryo transfer, gonadotropin‐releasing hormone agonist, persistent chronic endometritis, pregnancy outcomes

## Abstract

**Purpose:**

This study aimed to evaluate the pregnancy outcomes of frozen embryo transfer (FET) in patients with persistent chronic endometritis (PCE).

**Methods:**

186 patients were stratified into three groups: Cured CE (CCE), Cured Persistent CE (CPCE), and Still Persistent CE (SPCE). Only the first post‐treatment FET cycle per patient was analyzed.

**Results:**

Among women aged 20–48, the biopsy‐based prevalence of CE and PCE was 14.23% and 1.30%, respectively. The SPCE group underwent more antibiotic treatments and endometrial pathology examinations, with a higher proportion of GnRH‐a + HRT cycles compared to the CCE and CPCE groups. Clinical pregnancy rates were 41.67%, 37.21%, and 52.17%, while live birth rates were 31.67%, 30.23%, and 43.48% for the CCE, CPCE, and SPCE groups, respectively. No statistically significant differences were observed among the groups. Multivariate analysis confirmed that AMH (aOR 1.135; 95% CI 1.005–1.282) and pre‐transfer endometrial thickness (aOR 1.259; 95% CI 1.07–1.49) are significantly associated with clinical pregnancy.

**Conclusion:**

Our exploratory findings suggest that PCE may not necessarily impair FET outcomes under individualized management. Ovarian reserve and endometrial thickness appeared to be more robust predictors of success than histologic persistence in this cohort. Due to the small PCE sample size, these results are hypothesis‐generating and warrant validation in larger prospective trials.

**Trial Registration:**

This study is registered with China Medical Research Online (Registration Number: MR‐44‐24‐022293, www.medicalresearch.org.cn).

## 1 Introduction

Chronic endometritis (CE) is a persistent inflammatory disorder characterized by plasma cell infiltration within the endometrial stroma, frequently underdiagnosed due to its often asymptomatic clinical presentation. Epidemiological studies report a highly variable prevalence of CE among infertile populations (0.2%–56.8%), reflecting heterogeneity in study cohorts and diagnostic criteria [1]. Emerging evidence suggests CE as a significant contributor to female subfertility, with 14%–67.5% of patients experiencing recurrent implantation failure (RIF) demonstrating histopathological evidence of this condition [2].

The histopathologic features of CE include superficial edematous changes in the endometrium, high stromal cell density, dissociated maturation between the epithelium and stroma, and infiltration of endometrial stromal plasmacytes [3]. This pathognomonic characteristic is facilitated by complex pathogen‐host interactions [4]. Predominant microbial pathogens include *Ureaplasma spp*. (prevalence: 63.2%), *Chlamydia trachomatis* (31.6%), and *Mycoplasma spp*. (26.3%). These pathogens not only induce direct tissue injury but also trigger immune‐inflammatory cascades (e.g., NLRP3 inflammasome activation) via microbial components such as lipopolysaccharide (LPS), perpetuating chronic inflammation [5]. Furthermore, CE disrupts endometrial immune homeostasis, manifesting as aberrant immune cell distribution—elevated CD16^+^, CD20^+^, and CD56^+^ cell populations alongside diminished HLA‐DRII expression—suggesting autoimmune involvement in 90.6% of infertile CE cases [6]. This immune dysregulation perturbs the cytokine network, with excessive Th1/Th17‐associated pro‐inflammatory mediators (IL‐1β, IL‐18) and insufficient anti‐inflammatory responses sustaining a chronic inflammatory milieu [7]. The alarmin HMGB1 (high‐mobility group box 1 protein) serves as a pivotal mediator, where its overexpression induces macrophage pyroptosis, amplifying tissue damage and inflammation [8]. Collectively, this aberrant immune microenvironment impairs endometrial immune tolerance, compromising the finely regulated processes essential for successful embryo implantation [9].

Endometrial biopsy with histological examination remains the gold standard for diagnosing CE, with immunohistochemical detection of CD138 enhancing diagnostic sensitivity [10]. First‐line treatment typically consists of doxycycline (200 mg daily for 14 days), achieving a cure rate of 74.7% after one course and 89.7% after two courses, indicating antibiotic resistance in approximately 10% of CE patients [11, 12]. Currently, no consensus exists regarding the definition of persistent chronic endometritis (PCE). Some investigators define PCE as failure to achieve remission following a single antibiotic course, while others reserve this term for cases refractory to two treatment courses, alternatively classifying them as “refractory endometritis” [13]. Additionally, certain studies categorize cases unresponsive to three treatment courses as “multi‐drug resistant chronic endometritis” (MDR‐CE) [14]. A 10‐year longitudinal study involving over 3,000 women with recurrent implantation failure demonstrated an increase in MDR‐CE prevalence from 7% in 2010 to nearly 14% in 2021 [14]. The pathogenesis of PCE may involve antibiotic resistance, prior intrauterine procedures, retained gestational tissue, and dysregulated endometrial immune responses. Although multiple studies have investigated CE treatment outcomes, the optimal management of persistent cases remains uncertain, especially in enhancing frozen embryo transfer (FET) outcomes. This study intends to conduct a retrospective cohort analysis by enrolling PCE patients with different treatment statuses, analyzing core indicators such as embryo implantation rate, clinical pregnancy rate, live birth rate, and early miscarriage rate during FET cycles. Furthermore, it aims to explore the impact of factors such as FET endometrial preparation protocols and endometrial thickness on pregnancy outcomes, with the objective of providing scientific references for personalized treatment and FET strategy optimization in PCE patients.

## 2 Materials and Methods

### 2.1 Study Population

This investigation constitutes a retrospective cohort study conducted between January 1, 2018, and December 31, 2023. Histopathological evaluations, encompassing hematoxylin and eosin (H&E) staining and CD138 immunohistochemical analyses, were performed on endometrial specimens obtained via Pipelle biopsy catheter or hysteroscopy‐guided dilation and curettage procedures at our institution. The study focused on comparative analysis of FET outcomes among infertile patients undergoing assisted reproductive technology (ART) at our reproductive medicine center. The study population comprised women aged 20–48 years who underwent FET within 6 months post‐completion of antibiotic therapy for CE. Each patient contributed only their first FET cycle after therapy to the analysis. Exclusion criteria were established as follows: incomplete documentation regarding CE treatment protocols, patients undergoing fresh embryo transfer following CE management, presence of significant uterine anatomical abnormalities, and individuals with a history of endometrial hyperplasia or carcinoma treatment. Ethical approval for this study was granted by the Institutional Review Board of Sun Yat‐sen Memorial Hospital, Sun Yat‐sen University (Approval No. SYSKY‐2024‐354‐01). The study protocol was registered with the Chinese Medical Research Registration Information System (Registration No. MR‐44‐24‐022293; www.medicalresearch.org.cn).

### 2.2 Diagnostic Criteria and Treatment of CE

The diagnosis of CE remains challenging due to the absence of universally accepted diagnostic criteria. In this study, we employed the following criteria, where the presence of any one criterion was sufficient for CE diagnosis [11, 15]:

- Histological Evaluation (HE Staining Criteria): The presence of typical plasma cells in the endometrial stroma, either scattered or clustered in focal areas, is observed.
- Immunohistochemical Analysis: The presence of ≥ 5 CD138+ cells in one high‐power field (HPF;×400) within the endometrial stroma.

All endometrial biopsy specimens were processed and analyzed at a centralized laboratory by board‐certified pathologists with specialized expertise in endometrial pathology. For patients diagnosed with CE, post‐antibiotic treatment endometrial biopsies were performed to assess therapeutic response. The resolution of CE was defined by the absence of plasma cells in the endometrial stroma and fewer than 5 CD138 + cells per HPF, classified as cured chronic endometritis (CCE). Cases demonstrating uncured CE following an initial antibiotic course were designated as persistent chronic endometritis(PCE) [13, 16], warranting continued antibiotic therapy and subsequent endometrial evaluations.

**Figure 1:**
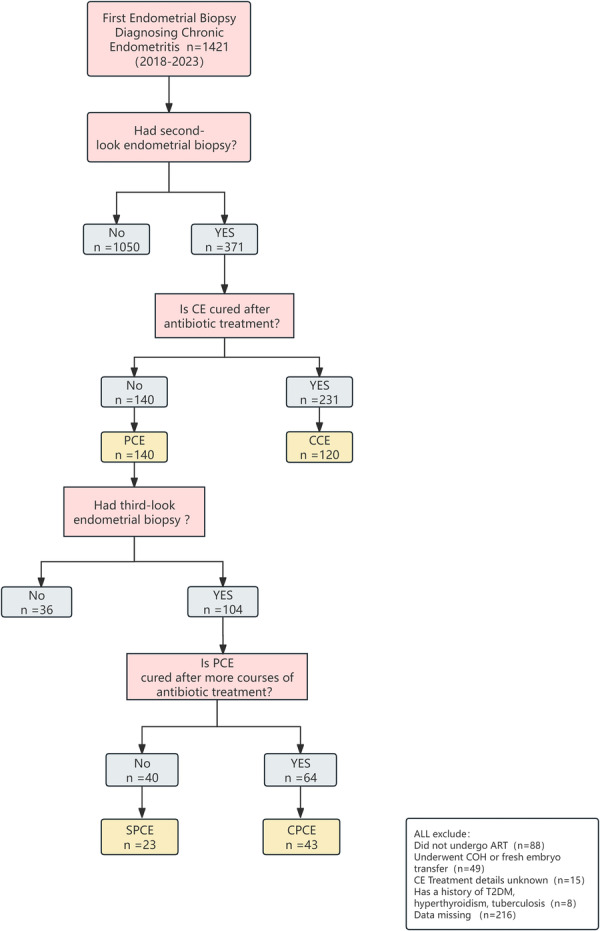
Diagram of the study and distribution of the patients investigated. CCE: cured chronic endometritis, CPCE: cured persistent chronic endometritis, PCE: persistent chronic endometritis, SPCE: still persistent chronic endometritis, TPCE: PCE who did not undergo a third endometrial pathological examination post‐treatment.

Prior to FET, patients were stratified into three diagnostic categories based on their most recent endometrial pathology findings: CCE, cured persistent chronic endometritis (CPCE), and still persistent chronic endometritis (SPCE). CPCE was defined by negative endometrial pathology results preceding FET, while SPCE encompassed patients with ongoing CE positivity. The study cohort comprised 186 participants, distributed as follows: 120 in the CCE group, 43 in the CPCE group, 23 in the SPCE group. In this study cohort, Doxycycline‐based regimens were the primary first‐line treatment, administered to 90.32% (168/186) of the patients. Other initial treatments included levofloxacin‐based (6.99%) or other antibiotic regimens (2.69%). Figure 1 shows the study flow.

### 2.3 Endometrial Preparation Protocol and Luteal Support

Endometrial preparation for FET cycles is individualized based on menstrual cycle and endometrial status, utilizing natural cycle, ovulation‐inducing cycle, HRT cycle, or GnRH‐a+HRT cycle. Endometrial thickness on the day of ovulation or progesterone initiation serves as the pre‐transfer assessment.

#### 2.3.1 Natural Cycle (NC)

Vaginal ultrasound monitoring begins on days 8–12 of the menstrual cycle to assess follicular development and endometrial status. When the dominant follicle exceeds 14 mm in diameter, urinary luteinizing hormone (LH) levels are closely monitored, along with serum follicle stimulating hormone (FSH), LH, estradiol and progesterone levels if necessary. If the dominant follicle reaches 18–20 mm with an endogenous LH peak (positive urinary LH test or serum LH > 20 U/L), the options include awaiting natural ovulation or administering a human chorionic gonadotropin (hCG) injection to induce ovulation. Day 0 is designated as the expected ovulation day, followed by luteal support post‐ovulation.

#### 2.3.2 Ovulation Induction Cycle(OI)

Ovulation induction typically begins on days 3–5 of the menstrual cycle. The treatment regimen involves daily administration of either tamoxifen (10–20 mg) or letrozole (2.5–5 mg) for five consecutive days. Based on follicular development, supplemental gonadotropins (Gn) may be required, with adjustments made according to ovarian response. When the dominant follicle reaches a diameter exceeding 20 mm and an endogenous LH peak is detected, ovulation is triggered by intramuscular injection of 10,000 U of hCG or subcutaneous injection of 250 μg of recombinant hCG (rhCG). This injection day is designated as Day 0 for ovulation.

Hormone Replacement Therapy Cycle(HRT): HRT begins on days 2–5 of the menstrual cycle with an oral dose of 6–8 mg of estradiol valerate daily, gradually adjusting the estrogen dosage based on endometrial growth. If needed, estradiol gel (2.5–5 g) can be applied topically twice daily. When progesterone is added to facilitate endometrial transformation, this day is designated as Day 0.

#### 2.3.3 GnRH‐a+HRT Cycle

GnRH‐a injections (3.75 mg or 1.87 mg) are administered on days 3–5 of the menstrual cycle or during the mid‐luteal phase. HRT is initiated 28 days later, with an estrogen regimen similar to that used in HRT.

Our center's luteal support protocol employs an individualized medication strategy, which can be broadly summarized as oral dydrogesterone at a dose of 10–20 mg taken twice daily (BID) combined with vaginal progesterone capsules 0.2 g administered three times daily (TID), or oral dydrogesterone combined with 40 mg of progesterone injection administered intramuscularly once daily (QD).

### 2.4 Embryo Grading

This study conducted embryo grading according to “Expert consensus on human embryo morphological assessment: cleavage‐stage embryos and blastocysts grading criteria” from the Chinese Association of Reproductive Medicine [17]. The grading system for cleavage‐stage embryos was classified into four levels (Grade I‐IV), with the evaluation primarily based on morphological parameters including blastomere number, fragmentation degree, blastomere uniformity, and multinucleation status. Blastocyst evaluation was performed according to the degree of expansion, hatching status, and inner cell mass morphology. This consensus, integrating the Vienna Consensus [18] and Gardner‐Schoolcraft blastocyst scoring system [19] with practical experience from multiple Chinese reproductive centers, defined high‐quality embryos as follows: for cleavage‐stage embryos ‐ Grade I/(8 cells), Grade IIa/(7–14 cells), and early fusion (≥ 7 cells); for day‐5 blastocysts ‐ expansion stage ≥ 3 with AA, AB, BA, or BB grading; and for day‐6 blastocysts ‐ expansion stage ≥ 4 with AA, AB, BA, or BB grading.

### 2.5 Pregnancy Outcomes

The primary outcome of this study was the clinical pregnancy rate. Patients underwent serum HCG testing 12–14 days post‐FET, with a positive result defined as a serum HCG concentration greater than 25 mIU/mL. The implantation rate was determined as the percentage of embryos that successfully implanted relative to the total number transferred. Clinical pregnancy was confirmed via vaginal ultrasound when at least one gestational sac was observed in the uterus 3–4 weeks following a positive HCG result. Early miscarriage was characterized by either the absence of a detectable heartbeat in the gestational sac or a spontaneous miscarriage occurring within the first 12 weeks of gestation. Live birth was defined as the delivery of an infant after the 28th week of gestation, with twins counted as a single event.

### 2.6 Statistical Analysis

Patient demographic characteristics and assisted reproductive technology (ART) treatment cycle data were extracted from the electronic medical record system, which comprehensively documents patient profiles, obstetric history, detailed FET cycle parameters, and treatment outcomes. Prior to database lock, all variables were checked for completeness. Cases with any missing values on the variables used in the analyses were excluded in their entirety. Consequently, no imputation or other missing‐data handling techniques were employed. Statistical analyses were conducted using R version 4.3.0 (R Foundation for Statistical Computing, Vienna, Austria), with graphical representations generated using Kingsoft WPS Office. Continuous variables exhibiting non‐normal distribution were expressed as median values with interquartile ranges (IQR; 25th–75th percentiles). Comparative analyses between two independent groups were performed using the Mann‐Whitney U test, while multiple group comparisons were conducted using the Kruskal‐Wallis H test. Categorical variables were presented as frequencies and percentages (*n*, %), with group comparisons analyzed using either the chi‐square test or Fisher's exact test, as appropriate. Binary logistic regression analysis was used to evaluate the impact of the other factors on the FET outcomes. *p* < 0.05 was considered statistically significant for all analyses.

## 3 Results

### 3.1 Biopsy‐Based Prevalence of Chronic Endometritis and Persistent Chronic Endometritis in the General Population From 2018 to 2023

**Figure 2:**
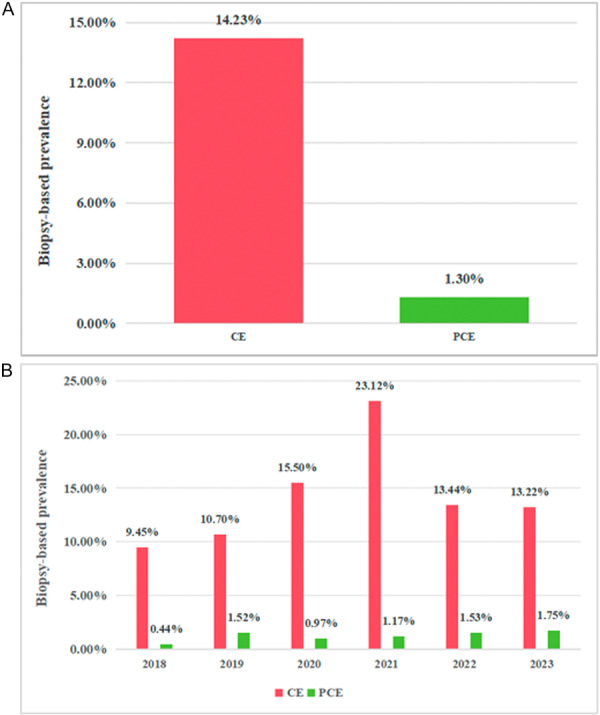
(A) Overall biopsy‐based prevalence of CE and PCE from 2018 to 2023; (B) Annual biopsy‐based prevalence of CE and PCE from 2018 to 2023.

Between 2018 and 2023, there were 11535 endometrial biopsies performed on women aged 20–48 years at our institution. Of these, 1,637 cases (14.23%) were diagnosed with CE, while 140 cases (1.30%) exhibited PCE (Figure 2A). As illustrated in Figure 2, the annual biopsy‐based prevalence of CE demonstrated temporal variability, peaking at 23.12% in 2021, with rates in other years remaining stable (10%–15%). In contrast, the biopsy‐based prevalence of PCE remained consistent initially but displayed an upward trend from 2021 onward, with rates of 1.17%, 1.53%, and 1.75% in 2021, 2022, and 2023, respectively (Figure 2B).

### 3.2 Comparative Analysis of Baseline Data Among CCE, CPCE, and SPCE

The baseline characteristics, including age, duration of infertility, anti‐Müllerian hormone (AMH) levels, body mass index (BMI), CA125 levels, types of infertility, history of intrauterine device (IUD) use, hydrosalpinx, and specific indications for ART, were found to be well‐balanced and statistically comparable across the CCE, CPCE, and SPCE groups. Notably, the SPCE group exhibited a significantly higher frequency of prior antibiotic treatments as well as a greater number of endometrial pathological examinations when compared to both the CCE and CPCE groups. These differences may reflect variations in clinical management or patient selection criteria specific to the SPCE protocol (Table 1).

**Table 1:** Baseline data among CCE, CPCE, and SPCE.

	CCE (*n* = 120)	CPCE (*n* = 43)	SPCE (*n* = 23)	Statistic (Z/χ²)	*p*
Maternal age (years)	34.00 (30.00,38.00)	34.00 (30.50,37.00)	33.00 (29.00,37.00)	0.36	0.834
AMH (ng/mL)	2.94 (1.43,5.06)	4.30 (2.40,5.80)	3.25 (1.53,5.05)	3.80	0.149
CA125 (mIU/mL)	15.55 (11.75,22.35)	18.60 (12.70,26.80)	17.50 (10.62,35.67)	1.70	0.427
BMI (kg/m^2^)	21.80 (19.50,24.00)	21.60 (19.95,23.35)	22.30 (20.40,23.77)	0.49	0.783
Type of infertility, *n*(%)				1.75	0.418
Primary	50 (41.67)	13 (30.23)	9 (39.13)		
Secondary	70 (58.33)	30 (69.77)	14 (60.87)		
Duration of infertility (years)	3.00 (2.00,6.00)	4.00 (2.50,6.50)	3.00 (2.00,5.00)	3.16	0.205
History of IUDs, *n*(%)	5 (4.17)	1 (2.33)	2 (8.70)	1.624	0.406
Hydrosalpinx, *n*(%)	25 (20.83)	5 (11.63)	6 (26.09)	2.48	0.289
Indications for ART				5.140	0.891
Fallopian tube factors	62 (51.67)	26 (60.47)	13 (56.52)		
Ovulation failure	15 (12.50)	5 (11.63)	5 (21.74)		
Endometriosis or Adenomyosis	4 (3.33)	2 (4.65)	1 (4.35)		
Male factors	14 (11.67)	5 (11.63)	2 (8.70)		
Combined	10 (8.33)	3 (6.98)	1 (4.35)		
Unexplained	15 (12.50)	2 (4.65)	1 (4.35)		
Course of antibiotics(*n*)	1.00 (1.00,1.00)	2.00 (2.00,2.00)	3.00 (2.00,3.00)	130.33	**< 0.001**
Number of endometrial biopsy(*n*)	2.00 (2.00,2.00)	3.00 (3.00,3.00)	3.00 (3.00,4.00)	167.69	**< 0.001**

*Note:* Data presented as median [25th percentile–75th percentile] or *n* (%).

Abbreviations: Z, Mann‐Whitney test; χ², Chi‐square test.

### 3.3 Comparative Analysis of FET Outcomes Among CCE, CPCE, and SPCE

Regarding endometrial preparation protocols for FET, the proportions of patients receiving gonadotropin‐releasing hormone agonist (GnRH‐a) combined with hormone replacement therapy (HRT) and other non‐GnRH‐a regimens were similar between the CCE and CPCE groups. However, a significantly higher proportion of patients in the SPCE group (78.26%) underwent the GnRH‐a + HRT protocol compared to the other two groups.

Statistical analysis revealed no significant differences among the groups regarding pre‐transfer endometrial thickness, number of transferred embryos, quantity of high‐quality embryos, or embryo types. In the FET outcomes, the implantation rates for CCE, CPCE, and SPCE groups were 28.42%, 26.87%, and 36.11%, respectively. The HCG positive rates were 51.67%, 44.19%, and 60.87%, while clinical pregnancy rates were 41.67%, 37.21%, and 52.17%. Additionally, live birth rates were 31.67%, 30.23%, and 43.48%, with corresponding early miscarriage rates of 24.00%, 12.50%, and 16.67% across the three groups (Table 2).

**Table 2:** FET outcomes among CCE, CPCE, and SPCE.

	CCE (*n* = 120)	CPCE (*n* = 43)	SPCE (*n* = 23)	Statistic (Z/*χ*²)	*p*(Unadjusted OR,95%CI)	
Endometrial preparation protocols, *n*(%)				14.54	0.024
NC	23 (19.17)	7 (16.28)	2 (8.70)			
HRT	30 (25.00)	10 (23.26)	2 (8.70)			
OI	13 (10.83)	10 (23.26)	1 (4.35)			
GnRH‐a+HRT	54 (45.00)	16 (37.21)	18 (78.26)			
Pre‐transfer endometrial thickness (mm)	10.00 (8.57,11.00)	10.00 (8.50,11.50)	10.00 (9.05,11.70)	1.55	0.461	
High‐quality embryos transferred (*n*)				0.40	0.983	
0	45 (37.50)	15 (34.88)	8 (34.78)			
1	45 (37.50)	17 (39.53)	8 (34.78)			
2	30 (25.00)	11 (25.58)	7 (30.43)			
Number and stage of embryo transferred, *n*(%)				2.190	0.984	
One Cleavage	13 (10.83)	5 (11.63)	1 (4.35)			
One Blastocyst	37 (30.83)	14 (32.56)	9 (39.13)			
Two Cleavage	47 (39.17)	15 (34.88)	10 (43.48)			
Two Blastocyst	19 (15.83)	8 (18.60)	3 (13.04)			
One Cleavage and one Blastocyst	4 (3.33)	1 (2.33)	0 (0.00)			
					**CCE vs CPCE**	**CCE vs SPCE**
Implantation rate, *n*(%)	54/190(28.42)	18/67 (26.87)	13/36(36.11)	1.06	0.807 (0.93, 0.49–1.73)	0.356 (1.42, 0.67–3.01)
HCG positive rate, *n*(%)	62 (51.67)	19 (44.19)	14 (60.87)	1.72	0.353 (0.74, 0.37–1.49)	0.420 (1.46, 0.59–3.62)
Clinical pregnancy rate, *n*(%)	50 (41.67)	16 (37.21)	12 (52.17)	1.39	0.610 (0.83, 0.41–1.70)	0.354 (1.53, 0.62–3.74)
Live birth rate, *n*(%)	38 (31.67)	13 (30.23)	10 (43.48)	1.39	0.862 (0.94, 0.44–1.99)	0.275 (1.66, 0.67–4.12)
Early miscarriage rate, *n*(%)	12 (24.00)	2 (12.50)	2 (16.67)	0.907	0.339 (0.45, 0.09–2.28)	0.593 (0.63, 0.12–3.30)

*Note:* Data presented as median [25th percentile‐75th percentile] or *n* (%).

Abbreviations: Z, Mann‐Whitney test; *χ²,* Chi‐square test.

### 3.4 Factors Influencing FET Outcomes: Binary Logistic Regression Analysis

Multivariable logistic regression analysis was conducted to assess factors associated with pregnancy outcomes in FET) The analysis incorporated variables identified through univariate binary regression and clinical relevance, including maternal age, AMH levels, BMI, pre‐transfer endometrial thickness, FET endometrial preparation protocol, number of transferred embryos, number of high‐quality embryos, and CEgroups (CCE, CPCE, SPCE).

For embryo implantation outcomes, pre‐transfer endometrial thickness demonstrated a significant positive association (OR 1.25; 95% CI 1.06–1.47; *p* = 0.007), indicating that increased endometrial thickness enhances implantation likelihood. AMH levels exhibited a marginally significant positive trend (OR 1.124; 95% CI 0.997–1.286; *p* = 0.055). Conversely, CE classification, endometrial preparation protocol, number of transferred embryos, number of high‐quality embryos, maternal age, and BMI did not reach statistical significance.

Clinical pregnancy outcomes closely mirrored implantation findings, with endometrial thickness remaining significantly beneficial (OR 1.259; 95% CI 1.07–1.49; *p* = 0.006). AMH levels were positively correlated with clinical pregnancy rate (OR 1.135; 95% CI 1.005–1.282;*p* = 0.042). CE groups showed a non‐significant directional trend. Transfer of a high‐quality embryo exhibited a borderline association with increased pregnancy likelihood (OR 1.642; 95% CI 0.979–2.753; *p* = 0.06), while other factors, including maternal age, BMI, and endometrial preparation protocols, remained non‐significant.

Among pregnancies that progressed, only advanced maternal age demonstrated a marginally negative association with live birth (OR 0.933; 95% CI 0.866–1.01; *p* = 0.066). However, pre‐transfer endometrial thickness, CE status, protocol selection, embryo quality, number of embryo transfer and BMI failed to demonstrate statistically significant predictive value in the final multivariate analysis (Table 3).

**Table 3:** Multivariable logistic regression to account for variables associated with FET pregnancy outcomes.

	Embryo implantation		Clinical pregnancy		Live birth	
	*P*	Adjusted OR (95% CI)	*P*	Adjusted OR (95% CI)	*P*	Adjusted OR (95% CI)
CE groups						
CCE	0.192	Reference	0.236	Reference	0.386	Reference
CPCE	0.105	0.488 (0.204 ~ 1.163)	0.132	0.511 (0.214 ~ 1.224)	0.606	0.795 (0.332 ~ 1.901)
SPCE	0.56	1.379 (0.468 ~ 4.067)	0.574	1.367 (0.46 ~ 4.057)	0.24	1.876 (0.657 ~ 5.354)
Endometrial preparation protocols						
NC	0.277	Reference	0.336	Reference	0.411	Reference
HRT	0.108	2.506 (0.817 ~ 7.682)	0.153	2.269 (0.738 ~ 6.98)	0.275	1.874 (0.607 ~ 5.786)
OI	0.563	1.452 (0.41 ~ 5.142)	0.679	1.31 (0.366 ~ 4.687)	0.836	0.87 (0.231 ~ 3.269)
GnRH‐a+HRT	0.888	1.072 (0.408 ~ 2.819)	0.995	1.003 (0.378 ~ 2.658)	0.843	0.903 (0.33 ~ 2.472)
Number of embryo transferred	0.846	0.927 (0.43 ~ 1.995)	0.63	0.825 (0.377 ~ 1.805)	0.695	1.174 (0.528 ~ 2.609)
High‐quality embryos transferred	0.081	1.577 (0.946 ~ 2.629)	0.06	1.642 (0.979 ~ 2.753)	0.202	1.396 (0.836 ~ 2.332)
Pre‐transfer endometrial thickness	0.007	1.25 (1.062 ~ 1.472)	0.006	1.259 (1.067 ~ 1.486)	0.336	1.081 (0.922 ~ 1.266)
Maternal age	0.237	0.957 (0.891 ~ 1.029)	0.305	0.963 (0.895 ~ 1.035)	0.066	0.933 (0.866 ~ 1.005)
AMH	0.055	1.124 (0.997 ~ 1.268)	0.042	1.135 (1.005 ~ 1.282)	0.333	1.057 (0.945 ~ 1.183)
BMI	0.102	1.094 (0.982 ~ 1.219)	0.098	1.096 (0.983 ~ 1.222)	0.222	1.072 (0.959 ~ 1.198)

## 4 Discussion

The prevalence of CE exhibits significant variation across different populations. The overall prevalence in healthy populations is approximately 10% [20], whereas in specific groups such as those with infertility, recurrent pregnancy loss (RPL), or recurrent implantation failure(RIF), the prevalence can reach as high as 24.4%–67.6%. This condition may be associated with various gynecological disorders including endometriosis, uterine fibroids, and endometrial hyperplasia. Among perimenopausal women, the overall CE incidence is 24.4%, with rates reaching 40.8% and 40.7% in patients with recurrent pregnancy loss and abnormal uterine bleeding, respectively [21]. The LEE study revealed a CE incidence of 24.7% in infertile women, with primary infertility, secondary infertility, RPL, and RIF subgroups showing incidences of 28.1%, 19%, 22.2%, and 27.2%, respectively [22]. Our study revealed an overall CE biospy‐based prevalence of 14.23% and in women of reproductive age. During the period from 2018 to 2023, the year 2021 demonstrated a significantly higher CE incidence compared to other years, potentially attributable to the impact of the COVID‐19 pandemic in 2020 on healthcare‐seeking behavior, followed by the gradual resumption of medical services and increased patient visits in 2021. Elena HogenEsch et al. reported a PCE prevalence of approximately 31% among CE patients [13], whereas our study found a prevalence of about 8.55% (140/1637) among CE patients. The overall incidence of PCE was approximately 1.3%, remaining relatively stable annually, though showing a slight upward trend after 2021 (from 1.17% to 1.75%). This increase may be related to factors such as improved diagnostic techniques, heightened awareness among healthcare providers and patients, and potential antibiotic overuse, though the precise causes warrant further investigation.

This retrospective analysis examined FET outcomes in patients with persistent chronic endometritis (PCE). We compared outcomes under three conditions: cured chronic endometritis (CCE), cured persistent chronic endometritis (CPCE), and still persistent chronic endometritis (SPCE). Among the three patient groups involved in this study, no significant differences were observed in baseline characteristics, embryo implantation rates, clinical pregnancy rates, or live birth rates following FET. Regression analysis revealed that maternal age, AMH levels, pre‐transfer endometrial thickness, and the number of high‐quality embryos were critical factors for the FET embryo implantation rate and clinical pregnancy rate.

As our study demonstrates, the biopsy‐based prevalence of PCE is relatively low, and the definitions of PCE vary across studies. Consequently, research on pregnancy outcomes related to PCE is scarce, and the conclusions drawn from such studies are inconsistent. A 2022 systematic analysis of RIF patients with CE showed that implantation rate (IR), clinical pregnancy rate (CPR), and ongoing pregnancy/live birth rate (OPR/LBR) were 20.7% (28/135), 35.2% (25/71), and 23.9% (17/71) for PCE patients, respectively. For CCE patients, these rates were significantly higher: 40.9% (169/413), 64.9% (144/222), and 41.4% (92/222). However, the analysis did not specify the diagnostic criteria for “PCE” or explore the treatment status of PCE patients [16]. In Yu Zhang's study, patients with CE who showed no improvement after one course of dexamethasone combined with antibiotic intrauterine infusion were defined as having PCE. Among these 24 PCE patients with RIF, no further antibiotic treatment was administered, and they proceeded directly to fresh embryo transfer, resulting in a clinical pregnancy rate of 25%, significantly lower than that of CCE patients at 51.76% (*p* = 0.02) [23]. Ettore Cicinelli's study found similar results: 15 RIF patients with PCE, after only one course of antibiotic treatment, had lower clinical pregnancy and live birth rates following fresh embryo transfer compared to patients without CE (33.0% vs. 65.2%, *p* = 0.039; and 13.3% vs. 60.8%, *p* = 0.02) [24]. Both studies focused on PCE patients who received just one course of antibiotics and analyzed outcomes in fresh cycles. The study from another team found that PCE patients who failed to improve after two courses of antibiotic treatment had lower embryo implantation rates, clinical pregnancy rates, and live birth rates during FET cycles compared to patients without CE. However, this research did not compare these patients with those who were cured of CE and only included natural cycles and HRT cycles, excluding GnRH‐a + HRT protocols [11]. In contrast to previous studies, our research stands out because both the CPCE and SPCE groups underwent at least two cycles of antibiotic treatment, alongside a variety of FET endometrial preparation protocols. This comprehensive approach may be a key factor in why the pregnancy outcomes in the CCE, CPCE, and SPCE groups were remarkably similar during FET cycles, highlighting the robustness and clinical relevance of our findings.

GnRH‐a induces ovarian suppression via pituitary downregulation, inhibiting FSH and LH secretion and reducing circulating estrogen and progesterone levels. Besides creating a hypoestrogenic state, some research shows it has anti ‐ inflammatory properties. Clinical investigations have revealed that the administration of GnRH‐a leads to a reduction in macrophage infiltration and endometrial microvascular density among patients with endometriosis [25]. Pre‐treatment with GnRH‐a can enhance the expression of IL − 6 and IL − 11 in endometrial stromal cells and augment endometrial receptivity [26]. These findings suggest GnRH‐a may have therapeutic effects through endocrine modulation and direct anti‐inflammatory mechanisms. Furthermore, GnRH‐a pre‐treatment might restore endometrial secretion of implantation‐related factors like HOXA10 and LIF(leukemia inhibitory factor). These factors are crucial for regulating endometrial development, promoting embryo implantation, and facilitating endometrium decidualization [27]. Therefore, for CE patients, particularly those with treatment‐resistant cases, some clinicians may adopt a GnRH‐a pre‐treatment strategy. The aim is to improve the inflammatory state of the endometrium, thereby enhancing its receptivity and increasing the success rates of FET. This therapeutic inclination is also reflected in our study, where the number of GnRH‐a + HRT cycles in the SPCE group was almost double that of the CPCE group (78.26% vs. 37.2%, *p* < 0.05). However, regression analysis revealed that the choice of FET endometrial preparation protocol had no significant impact on embryo implantation rate, clinical pregnancy rate, or live birth rate in patients with CCE, CPCE, or SPCE. However, due to the sample size in this study, it remains to be clarified through rigorous large‐scale randomized controlled trials (RCTs) whether GnRH‐a pre‐treatment provides benefits for PCE patients and which endometrial preparation protocols are most appropriate for this patient population.

The study found no significant differences in FET pregnancy outcomes among the three patient groups, and the endometritis status (CCE, CPCE, SPCE) had no significant impact on FET pregnancy outcomes. The research indicated that female AMH levels and endometrial thickness prior to embryo transfer may influence FET pregnancy outcomes in these CE patients. AMH serves as a sensitive indicator for assessing ovarian reserve. Elevated AMH levels are typically associated with younger age and favorable ovarian reserve, potentially indicating superior oocyte quality, which consequently enhances embryo quality and survival rates. Research demonstrates a positive correlation between AMH levels and clinical pregnancy rates in FET cycles, establishing AMH as an independent predictor for FET pregnancy outcomes [28]. Endometrial growth represents one of the estrogen‐induced morphological and functional changes involved in the preparation of the endometrium for embryo implantation. A thin endometrium adversely affects pregnancy outcomes in FET. A 2025 meta‐analysis demonstrated that in HRT cycles, endometrial thickness positively correlates with clinical pregnancy rates and live birth rates in FET. When the endometrial thickness is < 7 mm, clinical pregnancy rates and live birth rates decrease significantly. The study also found that when endometrial thickness reaches ≥ 6 mm, the risk of miscarriage declines markedly (OR 0.54, 95% CI: 0.29–0.99) [29]. An international multicenter cohort study analyzing 30,676 PGT‐A cycles demonstrated that in hormone replacement therapy cycles, when endometrial thickness was < 7 mm, the live birth rate significantly decreased (aOR 0.78, 95% CI 0.70–0.87, *p* ≤ 0.001), with a 22% reduction in the odds of live birth; in modified natural cycles, when endometrial thickness was < 7 mm, the live birth rate showed a more pronounced decline (aOR 0.59, 95% CI 0.49–0.72, *p* < 0.001), with a 41% reduction in the odds of live birth [30].

In the regression analysis of this study, the results regarding the number of high‐quality embryos were at the threshold level (embryo implantation rate: OR 1.577; 95% CI 0.946–2.629; *p* = 0.081; clinical pregnancy rate: OR 1.642; 95% CI 0.979–2.753; *p* = 0.06). These results suggest that embryo quality may exert a substantial influence on FET outcomes. This observation aligns with previous research, reinforcing the notion that the number of high‐quality embryos transferred serves as a critical determinant of clinical pregnancy rate, embryo implantation rate, and live birth rate in FET cycles. Moreover, optimizing the number of high‐quality embryos transferred may significantly enhance pregnancy success rates [31, 32]. For CE patients, particularly those with PCE, individualized embryo transfer protocols should be developed. In addition to the status of endometritis, comprehensive consideration should be given to factors such as AMH levels, endometrial thickness prior to transfer, and the number of high‐quality embryos available.

This study addresses an important and underexplored clinical question: whether persistent chronic endometritis (PCE) necessarily compromises FET outcomes, offering clinically relevant insights where literature is currently limited. A key distinction of our study is that the PCE cohort consists of patients who remained histologically positive after at least two intensive courses of antibiotic treatment, rather than being untreated cases. However, several critical limitations must be emphasized. First, the sample size of the still persistent CE (SPCE) group (*n* = 23) is relatively small, which inherently limits the statistical power to detect subtle differences and results in wide confidence intervals for our odds ratio estimates. As such, our findings must be regarded as strictly hypothesis‐generating rather than definitive evidence of clinical equivalence. Second, as a retrospective study, it may not fully capture all confounding factors; although we adjusted for variables like AMH and endometrial thickness, the lack of stratification by prior implantation failure history may introduce selection bias. Third, we utilized a diagnostic threshold of ≥ 5 CD138 + cells/HPF based on established literature [11, 15], though the lack of international diagnostic standardization remains a challenge for generalizability. Fourth, the heterogeneity of individualized interventions following multiple antibiotic failures makes it difficult to isolate the specific biological impact of histologic persistence. Finally, as a single‐center exploratory study, these results warrant validation in larger, prospective trials.

This study indicates a biopsy‐based prevalence of PCE of ~1.3% among women aged 20–48. Our exploratory findings suggest that patients with persistent chronic endometritis may achieve pregnancy outcomes similar to those of cured patients when managed with individualized protocols. While achieving a complete histological “cure” has traditionally been the primary clinical goal, our data indicate that other factors, such as ovarian reserve and endometrial thickness, may also play critical roles in determining FET success in this treated cohort. Given the limited sample size and retrospective constraints, clinicians should consider a holistic evaluation rather than a singular focus on “histologic cure” in cases of persistent disease. Further large‐scale prospective studies are essential to validate the safety and efficacy of this de‐emphasized approach.

## Author Contributions

Yihua Liang was responsible for conducting the research, collecting data, drafting the article, and analyzing the data. Zhaoyue Huang was responsible for drafting the article, analyzing the data, reviewing the manuscript. Haiyan Lin, Qi Qiu, and Xuedan Jiao were responsible for conducting the research and collecting data. Qingxue Zhang were responsible for critically reviewing the intellectual content of the article, securing research funding, and providing guidance.

## Ethics Statement

All procedures followed were in accordance with the ethical standards of the responsible committee on human experimentation (institutional and national) and with the Helsinki Declaration of 1964 and its later amendments.

## Consent

Informed consent was obtained from all patients for being included in the study.

## Conflicts of Interest

The authors declare no conflicts of interest.

## Declaration of Generative AI in Scientific Writing

During the preparation of this work, the authors used Deepseek in order to improve the English language and readability. After using this service, the authors reviewed and edited the content as needed and take full responsibility for the content of the publication.

## Data Availability

The data that support the findings of this study are available from the corresponding author upon reasonable request. The data are not publicly available due to privacy or ethical restrictions.

## References

[1] B. Geysenbergh , A.‐S. Boes , C. Bafort , et al., “The Impact of Chronic Endometritis on Infertility: Prevalence, Reproductive Outcomes, and the Role of Hysteroscopy as a Screening Tool,” Gynecologic and Obstetric Investigation 88 (2023): 108–115, 10.1159/000529304. 36739858

[2] F. Kimura , A. Takebayashi , M. Ishida , et al., “Review: Chronic Endometritis and Its Effect on Reproduction,” Journal of Obstetrics and Gynaecology Research 45 (2019): 951–960, 10.1111/jog.13937. 30843321

[3] K. Kitaya , T. Takeuchi , S. Mizuta , H. Matsubayashi , and T. Ishikawa , “Endometritis: New Time, new Concepts.” Fertility and Sterility (2018). 110, 344–350, 10.1016/j.fertnstert.2018.04.012. 29960704

[4] E. C. de Arruda Veiga , J. M. Soares Junior , M. Samama , et al., “Chronic Endometritis and Assisted Reproduction: A Systematic Review and Meta‐Analysis,” Revista Da Associacao Medica Brasileira 2023, no. 69 (1992): e20230792, 10.1590/1806-9282.20230792. PMC10578315 37851720

[5] S. Chen , L. Zhu , X. Fang , et al., “Alloferon Mitigates LPS‐Induced Endometritis by Attenuating the NLRP3/CASP1/IL‐1β/IL‐18 Signaling Cascade,” Inflammation 48 (2025): 730–746, 10.1007/s10753-024-02083-6. 38913143

[6] T. A. Demura , E. A. Kogan , Y. M. Kossovich , and A. L. Unanyan , “Morphological and Molecular Criteria of the Endometrial Reciptivity Violation in Patients With Infertility Associated With Chronic Endometritis,” VFSnegirev Archives of Obstetrics and Gynecology 5 (2018): 19–25, 10.18821/2313-8726-2018-5-1-19-25.

[7] M. L. Polina , L. M. Mikhaleva , I. I. Vityazeva , et al., “The Relationship Between the Microbiota Type and Immune Resources of the Endometrium Among Infertile Women in the Implantation Window Phase,” Yakut Medical Journal 82 (2023): 8–13, 10.25789/YMJ.2023.82.02.

[8] G. Yang , Q. Zhang , J. Tan , et al., “HMGB1 Induces Macrophage Pyroptosis in Chronic Endometritis,” International Immunopharmacology 123 (2023): 110706, 10.1016/j.intimp.2023.110706. 37541110

[9] S. Zeng , X. Liu , D. Liu , and W. Song , “Research Update for the Immune Microenvironment of Chronic Endometritis,” Journal of Reproductive Immunology 152 (2022): 103637, 10.1016/j.jri.2022.103637. 35576684

[10] Y. Liu , X. Chen , J. Huang , et al., “Comparison of the Prevalence of Chronic Endometritis as Determined by Means of Different Diagnostic Methods in Women With and Without Reproductive Failure,” Fertility and Sterility 109 (2018): 832–839, 10.1016/j.fertnstert.2018.01.022. 29778382

[11] Y. Xiong , Q. Chen , C. Chen , et al., “Impact of Oral Antibiotic Treatment for Chronic Endometritis on Pregnancy Outcomes in the Following Frozen‐thawed Embryo Transfer Cycles of Infertile Women: A Cohort Study of 640 Embryo Transfer Cycles.” Fertility & Sterility (2021). 116, 413–421, 10.1016/j.fertnstert.2021.03.036. 33926717

[12] E. Cicinelli , D. De Ziegler , R. Nicoletti , et al., “Chronic Endometritis: Correlation Among Hysteroscopic, Histologic, and Bacteriologic Findings in a Prospective Trial With 2190 Consecutive Office Hysteroscopies,” Fertility and Sterility 89 (2008): 677–684, 10.1016/j.fertnstert.2007.03.074. 17531993

[13] E. HogenEsch , R. Hojjati , A. Komorowski , et al., “Chronic Endometritis: Screening, Treatment, and Pregnancy Outcomes in an Academic Fertility Center,” Journal of Assisted Reproduction and Genetics 40 (2023): 2463–2471, 10.1007/s10815-023-02902-z. 37558906 PMC10504221

[14] K. Kitaya , S. E. Tanaka , Y. Sakuraba , and T. Ishikawa , “Multi‐Drug‐Resistant Chronic Endometritis in Infertile Women With Repeated Implantation Failure: Trend Over the Decade and Pilot Study for Third‐Line Oral Antibiotic Treatment,” Journal of Assisted Reproduction and Genetics 39 (2022): 1839–1848, 10.1007/s10815-022-02528-7. 35653041 PMC9428093

[15] Y. Li , S. Xu , S. Yu , et al., “Diagnosis of Chronic Endometritis: How Many CD138+ Cells/HPF in Endometrial Stroma Affect Pregnancy Outcome of Infertile Women?,” American Journal of Reproductive Immunology 85 (2021): e13369, 10.1111/aji.13369. 33152123

[16] Y. Zhang , H. Xu , Y. Liu , et al., “Confirmation of Chronic Endometritis in Repeated Implantation Failure and Success Outcome in IVF‐ET After Intrauterine Delivery of the Combined Administration of Antibiotic and Dexamethasone,” American Journal of Reproductive Immunology (2019): 82, 10.1111/aji.13177. 31373128

[17] D. Li and R. Li , “Expert Consensus on Human Embryo Morphological Assessment: Cleavage‐Stage Embryos and Blastocysts Grading Criteria,” Chinese Association of Reproductive Medicine 42 (2022): 1218–1225.

[18] The Vienna Consensus: Report of An Expert Meeting on The Development of ART Laboratory Performance Indicators . Reproductive Biomedicine Online. 35 (2017): 494–510, 10.1016/j.rbmo.2017.06.015. 28784335

[19] W. B. Schoolcraft , D. K. Gardner , M. Lane , T. Schlenker , F. Hamilton , and D. R. Meldrum , “Blastocyst Culture and Transfer: Analysis of Results and Parameters Affecting Outcome in Two In Vitro Fertilization Programs,” Fertility and Sterility 72 (1999): 604–609, 10.1016/S0015-0282(99)00311-8. 10521095

[20] D. B. McQueen , K. P. Maniar , R. Confino , L. Bernardi , and M. E. Pavone , “Chronic Endometritis: What Is The Prevalence Among Healthy Controls,” Fertility and Sterility 114 (2020): e45–e46, 10.1016/j.fertnstert.2020.08.147. 34120737

[21] D. Song , X. Feng , Q. Zhang , et al., “Prevalence and Confounders of Chronic Endometritis in Premenopausal Women With Abnormal Bleeding or Reproductive Failure,” Reproductive BioMedicine Online 36 (2018): 78–83, 10.1016/j.rbmo.2017.09.008. 29111313

[22] H. Lee , J. Kim , D. Choi , et al., “P‐319 Chronic Endometritis Prevalence, Associated Factors and Pregnancy Outcome Among Infertile Women,” supplement, Human Reproduction 39, no. S1 (2024): deae108.684, 10.1093/humrep/deae108.684.

[23] X. Cheng , Z. Huang , Z. Xiao , and Y. Bai , “Does Antibiotic Therapy for Chronic Endometritis Improve Clinical Outcomes of Patients With Recurrent Implantation Failure in Subsequent IVF Cycles? A Systematic Review and Meta‐Analysis,” Journal of Assisted Reproduction and Genetics 39 (2022): 1797–1813, 10.1007/s10815-022-02558-1. 35829835 PMC9428097

[24] E. Cicinelli , M. Matteo , R. Tinelli , et al., “Prevalence of Chronic Endometritis in Repeated Unexplained Implantation Failure and the IVF Success Rate After Antibiotic Therapy,” Human Reproduction 30 (2015), 323–330, 10.1093/humrep/deu292. 25385744

[25] K. N. Khan , M. Kitajima , K. Hiraki , et al., “Changes in Tissue Inflammation, Angiogenesis and Apoptosis in Endometriosis, Adenomyosis and Uterine Myoma After GnRH Agonist Therapy,” Human Reproduction 25 (2010), 642–653, 10.1093/humrep/dep437. 20008888

[26] L. Li , L. Liu , Z. Kou , M. Huo , J. An , and X. Zhang , “GnRH Agonist Treatment Regulates IL‐6 and IL‐11 Expression in Endometrial Stromal Cells for Patients With HRT Regiment in Frozen Embryo Transfer Cycles,” Reproductive Biology 22 (2022): 100608, 10.1016/j.repbio.2022.100608. 35151984

[27] B. Xu , D. Geerts , S. Hu , et al., “The Depot Gnrh Agonist Protocol Improves the Live Birth Rate Per Fresh Embryo Transfer Cycle, but not the Cumulative Live Birth Rate in Normal Responders: A Randomized Controlled Trial and Molecular Mechanism Study,” Human Reproduction 35 (2020): 1306–1318, 10.1093/humrep/deaa086. 32478400

[28] S. Vagios , J. Y. Hsu , C. R. Sacha , et al., “Pretreatment Antimüllerian Hormone Levels and Outcomes of Ovarian Stimulation With Gonadotropins/Intrauterine Insemination Cycles,” Fertility and Sterility 116 (2021): 422–430, 10.1016/j.fertnstert.2021.02.047. 33823994

[29] F. Pérez‐Milán , M. Caballero‐Campo , M. Carrera‐Roig , et al., “Impact of Endometrial Thickness on Reproductive Outcome in Fresh and Frozen–Thawed Embryo Transfer: Systematic Review and Meta‐Analysis,” Ultrasound in Obstetrics & Gynecology: The Official Journal of the International Society of Ultrasound in Obstetrics and Gynecology 66 (2025): 271–281, 10.1002/uog.29270. 40757788 PMC12401503

[30] H. Genovese , C. A. Mayo , E. Kalafat , et al., “Does Endometrial Thickness Impact Live Birth Rate Following a Frozen Embryo Transfer: Outcomes of 30 676 Euploid Single Embryo Transfers,” Human Reproduction 40 (2025): 1919–1927, 10.1093/humrep/deaf129. 40639807

[31] J. Wang , Z. Yang , Y. Chen , et al., “Analysis of Factors Influencing Clinical Pregnancy Rates in Frozen‐Thawed Embryo Transfer Cycles,” Frontiers Endocrinology 16 (2025): 1551530, 10.3389/fendo.2025.1551530. PMC12237683 40636712

[32] W. Gao , W. Tang , C. Li , et al., “Effect of Maternal Age, Embryo Number and Quality on Pregnancy Outcome During Frozen Embryo Transfer Cycle,” Frontiers Endocrinology 16 (2025): 1596178, 10.3389/fendo.2025.1596178. PMC12634337 41282292

